# Robotic Ivor-Lewis esophagectomy – How-we-do-it

**DOI:** 10.1016/j.sopen.2025.07.006

**Published:** 2025-08-05

**Authors:** Henrik Nienhüser, Nicolas Jorek, Ali Majlesara, Frank Pianka, Arianeb Mehrabi, Christoph W. Michalski

**Affiliations:** a Heidelberg University Hospital, Department of General, Visceral and Transplantation Surgery, Heidelberg, Germany

## Introduction

Esophageal cancer remains one of the most common causes of cancer-related mortality worldwide [1]. Resection of esophageal cancer is one of the most challenging procedures in gastrointestinal and thoracic surgery and requires a combination of abdominal and thoracic resection [2]. With the introduction of minimal-invasive techniques, particularly by establishing robotic-assisted procedures, the morbidity and mortality of Ivor-Lewis resection significantly decreased [3]. In the past, several techniques in robotic-assisted surgery have been described and investigated. In this video manuscript, we describe our standard for a robotic Ivor-Lewis resection in detail.

### Patient placement and port set-up

Patients are positioned in a supine split-leg position (French-position) in anti-Trendelenburg (16°) on a vacuum mattress. Capnoperitoneum (12 mmHg) is introduced via Veres-needle at Palmer's point and laparoscopy is performed through a 12 mm single-port in the left lower quadrant. Port #2 (8 mm) is placed slightly left to the umbilicus, Port #3 (8 mm) and #4 (8 mm) are placed 8 cm each to the left. Port #1 (12 mm) is placed about 10 cm to the right and can be used for stapling if needed. A second 12 mm assistance port is placed in the lower right quadrant. A liver retractor is inserted via a 5 mm port under the right rip arche before docking to the robot. Bipolar forceps are inserted in arm 1, while arm 3 is used for the scissors/vessel sealer and arm 4 is used for the tip-up forceps.

### Gastrolysis

The gastrolysis phase is started by incision of the gastrocolic ligament and opening of the lesser sac. This step is completed along the greater curvature and the splenic hilum with dissection of the short gastric vessels until the left crus of the diaphragm is reached. Subsequently, the gastrolysis is completed by mobilizing the stomach down to the pylorus respectively the origin of the gastroepiploic artery arising from the gastroduodenal artery. Finally, the greater curvature of the stomach is freed completely and remaining retrogastric adhesions are dissected.

### Lymphadenectomy celiac trunc

For central lymphadenectomy, the mobilized stomach is lifted to the ventral abdominal wall with the tip-up forceps (port #4) and the help of the table assistant. The left gastric vein is identified and dissected centrally using hemo-loc-clips. The hepatic artery is cleared up to the hepatoduodenal ligament. The left gastric artery is dissected using hemo-loc-clips as well. The remaining lymphadenectomy can be performed using the VesselSealer until the posterior commissure of the hiatus is completely dissected. Lymphadenectomy is completely performed in station 15 (left gastric artery) and station 16 (celiac trunc) [4]. Station 17 (splenic artery), station 18 (hepatic artery), and station 19 (hepatoduodenal ligament) are only dissected if clinically suspicious lymph nodes are present.

### Lymphadenectomy hiatus

The omentum minus is opened and the right crus of the diaphragm is exposed. By connecting the dissection lines from the greater and lesser curvature, the lymphadenectomy in the posterior part of the hiatus is completed until the ventral wall of the aorta is fully visible. A thorough dissection of the stations 13 L/R and 14 L/R is performed. The thoracic duct is either identified in this step and clipped using hemo-loc-clips or is closed later during the thoracic part of the operation. The ventral border of the lymphadenectomy is the pericard, the right pleura is opened after the dissection of the hiatus is complete. At this step, intraoperative discussion with the anesthesia team is crucial as the intraabdominal and intrathoracic pressure can be shifting at this point. The left pleura is usually preserved unless oncological reasons require a complete dissection. At the end of this phase, the stomach is fully mobile from pylorus to hiatus. The right crus is divided for better mobility of the gastric conduit and to avoid potential impression after the gastric pull-up. In case of an existing hiatal hernia, this step may be skipped.

### Formation of the gastric conduit

The stomach is put back in its original position to prevent malrotation and problems in gastric tube formation. For stapling, either robotic staplers or laparoscopic staplers can be used. We prefer a laparoscopic stapling device. The first 45 mm stapler is placed by the assistant within the gastric corpus at the lesser curvature. The conduit formation is started on the lower curvature about 3–4 cm from the pylorus (see Supplement No. 1). We prefer a relatively small width of the gastric conduit of around 3-4 cm. This can be measured using the robotic instruments. The gastric tube formation is completed by using 4–5 linear stapler magazines (60 mm) up to the fundus while leaving the final 3-4 cm of fundus intact. Then, the locations of double-stapling are inverted using 2–0 vicryl. A cholecystectomy is routinely performed in patients at risk for postoperative cholecystitis. A Robinson drain (16Ch) is inserted via the right assistant port and placed along the upper pancreatic margin into the hiatus.

### Patient placement, port set-up and mini-thoracotomy for thoracic phase

The patient is placed in a left lateral decubitus position within the vacuum mattress. Four robotic ports are placed. The first port (12 mm) is placed just over the 12th rib and three 8 mm ports are inserted in a distance of 8 cm from each other. The fourth port should be inserted one rib space above a line between the mamilla and the lower tip of the scapula. An assistant port is placed in this line and can be extended for the thoracotomy (Supplement No. 2). Arm 1 is used for the bipolar forceps, while arm 3 is used for scissors/vessel sealer and arm 4 for the tip-up forceps.

### Preparation of V. azygos and paraaortic lymphadenectomy

After exploration of the thoracic cavity and the start of the single-lung ventilation, the azygos vein is isolated and ligated by hem-o-lock clip.

Paraaortic lymphadenectomy is performed clearing lymph node stations 10R/L, 11R/L and 12R/L while stations 6–9 are only cleared in case of suspicious lymph nodes [4]. Paraaortic lymphadenectomy is performed using the vessel sealer. The abdominal resection is completed and the esophagus is fully mobilized to the level of the azygos vein.

### Cut-down esophagus

The esophagus is isolated just below the azygos vein and the vagal nerves are dissected. After this, the esophagus is cut down using scissors and a frozen section is sent from the distal margin.

### Creation of the esophago-gastrostomy

A purse string suture is performed (0 prolene, 20 cm). A mini-thoracotomy is performed in the location described above and a wound protector is inserted. The circular stapler anvil (29 mm) is placed in the esophageal stump by the assistant. After knotting of the purse string suture, the gastric conduit is pulled up into the thoracic cavity using the vicryl sutures to avoid manipulation to the conduit. The stapler is inserted via a gastrostomy into the specimen and the spike is turned out on the front side of the gastric conduit. The anvil is connected and the anastomosis is created. We prefer a powered-driven circular stapler for this step. Finally, the specimen is divided from the gastric conduit using a linear stapler inserted via the port #1 (12 mm). A nasogastric tube is put in under visual control and a thoracic drain (24Ch) is put in via port 1 and placed close to the anastomosis. A routine pyloric dilatation is not performed.

## Summary

In this manuscript, we describe our standardized technique of a robot-assisted Ivor-Lewis resection in detail. By the implementation of this step-by-step approach, this procedure can be taught safely using a dual-console approach.

## CRediT authorship contribution statement

**Henrik Nienhüser:** Writing – review & editing, Writing – original draft, Visualization, Validation, Project administration, Conceptualization. **Nicolas Jorek:** Writing – original draft, Visualization, Investigation, Conceptualization. **Ali Majlesara:** Writing – review & editing, Visualization. **Frank Pianka:** Writing – review & editing, Writing – original draft, Validation, Supervision. **Arianeb Mehrabi:** Writing – review & editing, Validation, Supervision, Project administration, Conceptualization. **Christoph W. Michalski:** Writing – review & editing, Supervision, Project administration, Conceptualization.

## Ethical approval

No ethics approval was needed for this article.

## Funding

The authors received no funding for the assembly of this manuscript.

## Declaration of competing interest

The authors declare no conflict of interest.

## Appendix A Supplementary data

Supplementary figures Image 1

Video S1 Robotic esophagectomy How we do it final abdominal phase. Video S1

Video S2 Robotic esophagectomy How we do it final thoracic phase. Video S2
