# Supplementary material for: Robotic Ivor-Lewis esophagectomy – How-we-do-it
Source: Surg Open Sci. 2025 Aug 5;27:104–5. doi: 10.1016/j.sopen.2025.07.006 (PMC12355179; doi:10.1016/j.sopen.2025.07.006)
Supplement: Supplementary file 1 — Supplementary figures [file mmc1.docx]

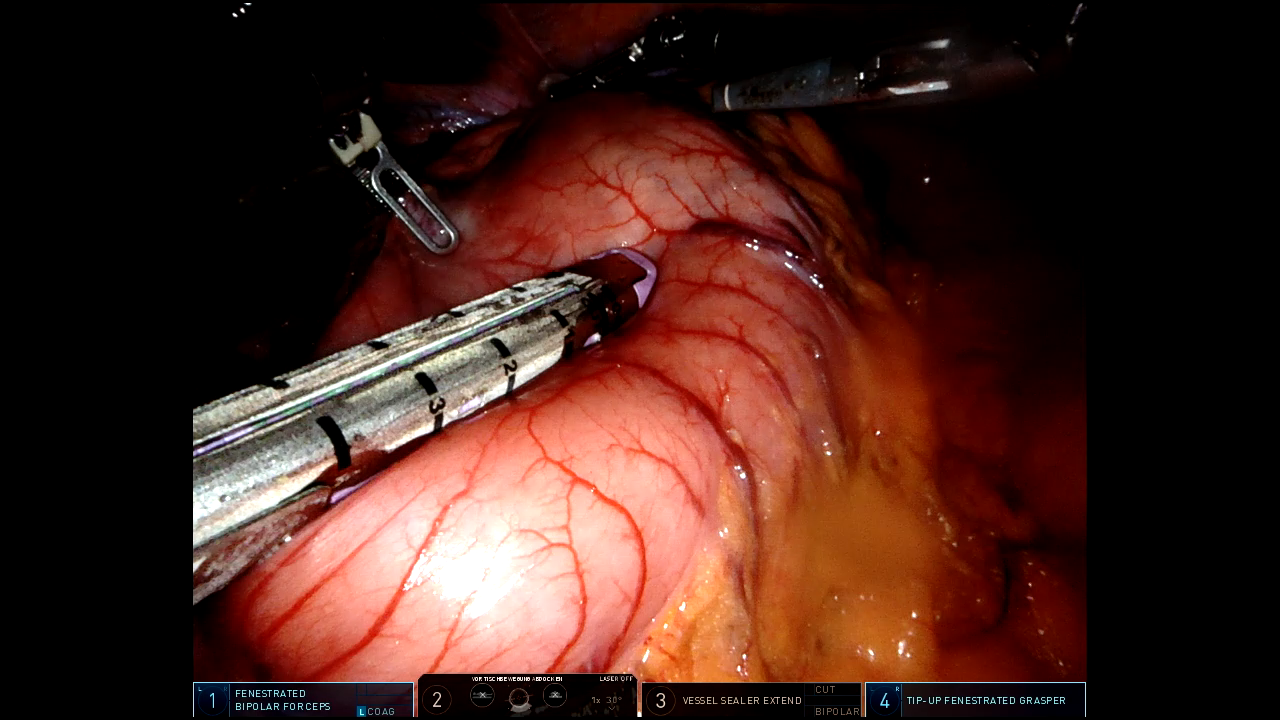


Supplement 1: Start of the conduit formation on the lower curvature


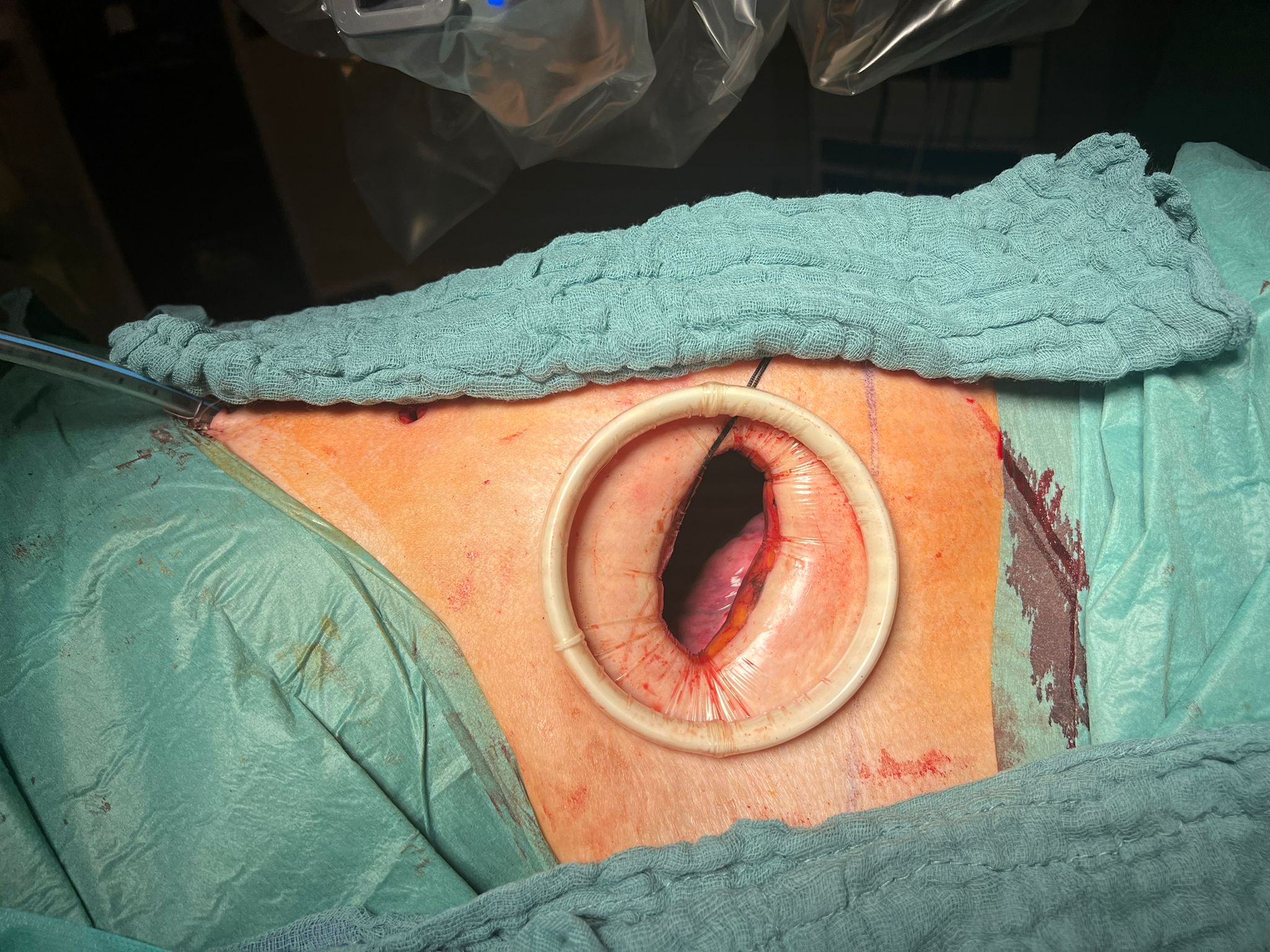


Supplement 2: Mini thoracotomy for insertion of the circular stapler and removal of the specimen after resection
